# New Perspectives for the Consolidation of Mural Paintings in Hypogea with an Innovative Aqueous Nanolime Dispersion, Characterized by Compatible, Sustainable, and Eco-Friendly Features

**DOI:** 10.3390/nano13020317

**Published:** 2023-01-12

**Authors:** Sara Iafrate, Giancarlo Sidoti, Filippo Edoardo Capasso, Manuel Giandomenico, Sokol Muca, Valeria Daniele, Giuliana Taglieri

**Affiliations:** 1 Istituto Centrale per il Restauro Ministero della Cultura (ICR), Via di San Michele 25, 00153 Roma, Italy; 2 Restorer and Conservator of Cultural Heritage, Sokol Muca, Via Giuseppe Verdi 5, 13100 Vercelli, Italy; 3 Department of Industrial and Information Engineering and Economics, University of L’Aquila, Piazzale E. Pontieri 1, 67100 Monteluco di Roio, L’Aquila, Italy

**Keywords:** Ca(OH)_2_ nanoparticles, nanomaterials in cultural heritage, hypogeum mural paintings, consolidation treatment, aqueous nanolime

## Abstract

Consolidation of mural paintings in hypogea is challenging because of their severe microclimatic conditions, characterized by high humidity levels, low air circulation, the presence of salts efflorescence, and the detrimental growth of biodeteriogen agents. Traditional consolidant products show significant drawbacks when used in hypogeum. Organic compounds, such as acrylic emulsions, are bio-receptive and some inorganic consolidants, such as silica-based products, show a lack of compatibility with the original substrate, which could lead to a reduction in permeability and an increase in the mechanical resistance of the external layer. The presence of solvents in their formulations, particularly short-chain alcohols that can activate germination of fungal spores, leads to the release of great amounts of volatile organic compounds, which are particularly harmful in the hypogeic environment. To solve these problems, restorers of the Istituto Centrale per il Restauro (ICR) decided to use a new aqueous nanolime dispersion, NANOLAQ, consisting of pure and crystalline Ca(OH)_2_ nanoparticles dispersed in water, produced by an innovative and sustainable patented procedure. After laboratory testing, the product has been applied on site, on a medieval mural painting in the Ss. Peter and Paul hypogeum in the UNESCO site of Matera (Italy), monitoring the performance in terms of cohesion of the paint layer and preservation of aesthetic features.

## 1. Introduction

Cultural hypogea represent unique and valuable sites of world heritage thanks to the great interest coming from their historical artistic and religious impacts. Hypogea are usually characterized by the presence of both stone materials and wall paintings of interest from historical, artistic, and religious points of view. Moreover, there are peculiar environmental conditions related to a specific microclimate generated by high humidity and low light conditions and air circulation. In fact, hypogea are often dark places with a high humidity level and strong thermal inertia. These environmental conditions create a favorable ambient for the growth of certain types of microorganisms, such as streptomyces, bacteria, and fungi [1,2]. The alteration of their microclimatic stability, due to air circulation or to visitors’ presence, could represent a further cause of deterioration [3]. When the environment is altered, evaporation processes can occur and determine superficial salt crystallization. Besides microclimatic instability, the permanence of visitors induces other damaging effects. In fact, visitors’ exhalations increase the carbon dioxide concentration, becoming carbonic acid in the presence of water, and induce the degradation of calcium carbonate substrate. In addition, visitors are vehicles of additional spores and microbiological contaminants, which negatively affect the hypogeum surfaces [4,5,6].

Definitively, to date, hypogea are extremely difficult sites for restoration and conservation of the artworks present inside them: this unique environment requires specific solutions, including an appropriate choice of the consolidation intervention.

The ideal consolidant for mural paintings in hypogeum should be, as much as possible, compatible with the original materials, dissolved or dispersed in solvents not harmful for the original materials, and especially for operators because the intervention is carried out in an environment with very low air circulation. These things considered, it should not alter the permeability of the surface to vapor and liquid water, it should be stable at high humidity conditions, and it should not be bio-receptive.

One of the main issues of the restoration intervention in hypogea is to select a suitable consolidant product possessing all the requirements mentioned above. This was a factual problem to such an extent that, in the 1950s, when the availability of consolidant products was limited to few materials, Cesare Brandi claimed that the only way to preserve damaged rupestrian hypogenic mural paintings in South Italy was to detach them from their original stone support and take them away from their severe environment [7,8]. This radical statement was based on the observed incompatibility and inefficiency of most of the consolidant products available at that time. Since the 1960s, synthetic resins have been employed to consolidate wall paintings [9]. In the hypogeic context [10], organic resins were extensively used for this kind of purpose because of their easy handling and quick setting. However, the use of these type of consolidants is detrimental because they form a coating that limits the vapor and water exchanges between the environment and the painted layer, inducing the crystallization of soluble salt under the paint layer and consequent mechanic tensions which lead to cracks and the disgregation of the original carbonatic substrate [11]. Furthermore, organic compounds are an ideal substrate for the growth of biodeteriogen agents [12]. Inorganic silica-based products have been also used as consolidants for mural paintings. However, even if they are less bio-receptive than organic compounds, conversely, they show very low mechanical and chemical compatibility, very low penetration in humid environments, and a tendency to form rigid and unaesthetic superficial encrustations [13]. In the early 2000s, nanolime consolidants in alcoholic suspension were introduced in the restoration of mural paintings [14,15]. Considering that high humidity can allow a complete carbonatation process [16], nanolimes present a perfect compatibility with the original carbonatic plasters, without significantly altering the porosity and hydrophily of the treated surface [17,18,19,20,21]. For these reasons, nanolime products have been tested in some hypogea in order to treat defects of cohesion of the painted layer [22,23]. However, despite their compatibility and low modification of the porosity of the painted treated surface, when applied on mural paintings in the hypogeic environment, the alcoholic nanolimes have considerable drawbacks: at high humidity conditions, the alcoholic nanolimes showed the formation of different mineralogical forms of calcium carbonate, that is calcite but also aragonite, as a result of the carbonatation process [16,24]. The short-chain alcohols in which they are dispersed not only could activate the germination of fungal spores [25], but they could also represent a risk of considerable toxicity for operators and visitors in such a low air exchange rate environment due to the release of many volatile organic compounds (VOCs) in the ambient. So that, the use of the alcoholic nanolimes does not represent an ideal solution for consolidation treatments in hypogea.

In order to make up for the lack of effective consolidant products in these specific and severe environmental conditions, the restorers of the Istituto del Restauro in Rome (ICR) decided to test a new product on a mural painting in the hypogeum of the Ss. Peter and Paul Church in the UNESCO site of Matera (Italy) during the ICR restoration intervention. This new product, which ICR restorers considered potentially suitable to all the conservative needs of the hypogeic environment, consists of the aqueous nanolime suspension NanoLAQ, obtained by an innovative and sustainable patented process developed at the University of L’Aquila [26,27]. Actually, the NanoLAQ suspension is constituted only by calcium hydroxide (Ca(OH)_2_) nanoparticles dispersed in water and, as previously published, it is characterized by the following features [20,21,28]: it is fully compatible with the carbonated substrate thanks to a complete transformation in pure calcite during the carbonatation process; it does not significantly alter the porosity of the treated substrates, leaving unaltered the permeability of the surface to water and vapor. In addition to all these features, being the dispersing solvent of the Ca(OH)_2_ nanoparticles water, germination of biodeteriogen agents is not triggered and the health of operators and visitors is preserved.

The present work is an applied case study aimed at presenting the preliminary results obtained by the use of the aqueous nanolime dispersion, employed, for the first time, for the consolidation of mural paintings in the hypogeum environment. Specifically, this new product has been applied on mural paintings in the Ss. Peter and Paul hypogeum in Matera, an UNESCO site in the south of Italy. Before application, the suspension has been analyzed in terms of chemical and phase composition and morphological features by X-ray diffraction (XRD), scanning and transmission electron microscopy (SEM and TEM, respectively). The optimization and the effectiveness of the nanolime treatment has been studied, carrying out different procedures to define the best application both in laboratory samples and on sample areas of the paintings directly on site.

In addition, a new method for testing the efficacy of the treatment, both on specimens and in situ, is here presented, aimed at enhancing the repeatability and reliability of standard procedures.

## 2. Materials and Methods

### 2.1. The Mural Paintings in the Hypogeum of Ss. Peter and Paul in Matera

Ss. Peter and Paul hypogeum in Matera is a cave site made of three underground rooms entirely carved in stone, in which some noticeable medieval mural paintings, dating between the XIII and XIV centuries A.D., are preserved. The hypogeum, which originally was probably a rupestrian church, is now located under the Saint Francis’ church, whose original structure dates to the XIII century. The only entrance to the crypt is a narrow manhole on the floor of one of the side chapels of the church, which connects the two spaces through a steep stair. The underground location and its isolation from the upper environment (the church) defines this site as a hypogeum [29]. The microclimatic survey revealed that this site has very high humidity levels year-round, ranging from 92% to 99% of relative humidity (R.H.), as expected in a typical hypogeum. Due to the high thermal inertia, no significant seasonal changes have been detected. The CO_2_ concentration is similar to that of the upper church, and it tends to increase with the presence of the visitors [30]. Humidity of the ambient, biodegradability of the materials, and increase in the CO2 concentration are the difficulties that affect the conservation treatment, as observed in all rupestrian hypogea [31,32,33,34].

The restoration intervention was carried out on two of the fresco paintings of the main room, and it represents a part of a thesis work carried out at the Istituto Centrale per il Restauro (ICR) [30]. The frescoes consist of a single layer of plaster made by air lime binder and calcareous aggregates. The average thickness of the plaster is 1 cm. The pictorial layer, whose thickness is about 200 µm, consists of few colors, mainly including earth pigments and white wash. The plaster is directly applied on the carved stone. The painted surface was partially hidden by salt concretions. The cleaning operation has been carried out to remove concretions on fresco surfaces, preferring mechanical tools and laser technology to chemical cleaning. After removing most of the concretions, the discovered pictorial layer showed lack of cohesion and adhesion, so that it needed a proper consolidation treatment before concluding the cleaning procedure.

### 2.2. Characterization of the Aqueous Nanolime Dispersion

The aqueous nanolime dispersion used in this work is a new commercially available nanolime, NanoLAQ, which is produced by a sustainable, high-yield, and eco-friendly process, based on an ion exchanges process, occurring in water and at room temperature in a single step between a calcium chloride aqueous solution and an anion exchange resin, according to a patented procedure described in previous papers [26,27,28,35].

In relation to previous results on natural stones [28], two different aqueous nanolime concentrations were considered, 10 g/L and 20 g/L, from here called NanoLAQ10 and NanoLAQ20. In addition, since the NanoLAQ suspension allows for the change in the solid/liquid concentration, a 300 g/L dispersion was considered, NanoLAQ 300, to treat defects of adhesion between the paint layer and the plaster.

The compositions of the NanoLAQ aqueous dispersions were analyzed in terms of structural and morphological features of the solid phase. Specifically, the solid phase was analyzed by X-ray diffraction (XRD, *PANAlytical X’Pert Pro*), scanning electron microscopy (FESEM Gemini SEM 500, ZEISS, *Oberkochen, Germany*), and transmission electron microscopy (TEM CM100, *Philips CM100, Amsterdam, the Netherlands*). All the investigations on the solid phase were carried out by drying a representative sample of the suspension under nitrogen, in order to avoid the carbonatation process. In particular, regarding the XRD patterns, 0,2 mL of suspension was taken and deposited to a zero-background sample holder. Once dried, the XRD pattern was acquired by a step scan, in the angular range 5–70° 2θ, elaborated by the HighScore Plus software package (PANalytical, Almelo, the Netherlands), and compared with ICSD (ICDD, Philadelphia, PA, USA) and ICDD (FIZ Karlsruhe GmbH, Eggenstein-Leopoldshafen, Germany) reference database for the qualitative analysis. The microscopic observations were carried out, under nitrogen atmosphere, as follows: for SEM analysis, about 1 mL of sample was left to dry on a SEM specimen stab; for TEM investigations, few drops of the diluted suspension were deposited on a suitable grid. 

Finally, before application, the chloride concentration was checked, by means of an ion-sensitive electrode (*Metrohm AG*, Herisau, Switzerland). In fact, the accuracy of interventions on mural paintings needs a controlled chemical composition of the employed products. In accordance with the specific request of the ICR restorers, the NanoLAQ dispersions were characterized by a concentration of 15 mg/l of chloride ions.

### 2.3. Tests Performed for the Evaluation of Treatment Efficacy on the Paint Layer

The action of the NanoLAQ suspensions was assessed both in terms of consolidating efficacy and in relation to its ability to avoid surface changes in the painted layer after the application. For these aims, the following tests were performed before and after the consolidation treatment:

− A: measurement of the variation in the ions’ content of the surface;
− B: pH measurement for the evaluation of the carbonatation degree over time;
− C: efficacy test of the treatment, in terms of superficial cohesion of the paint layer;
− D: colorimetric measurement to analyze alterations in the aesthetical coloring of the treated painted layers.

The study was conducted at first in laboratory, in order to individuate the best application procedure for the in situ treatment, assuring the required consolidation degree without altering any characteristic of the painted layers. For each test, specific specimens were prepared, simulating the composition of original plaster to satisfy the test requirements. In particular, for the ions’ content tests (A), mortar samples (5 × 5 × 1) cm^3^ in volume were prepared by mixing air lime and calcareous aggregates (travertine powder, grain size 0–0.7 mm), according to a 1:2 ratio, and cured for 28 days. In the case of pH and carbonatation tests (B), fresco specimens were prepared as follows: a layer of mortar, 1 cm thick, was spread on a brick. The composition of the mortar was identical to that prepared for A tests: air lime and calcareous aggregate (grain size 0–0.7 mm) in a 1:2 ratio. For this test, however, during the curing time, a layer of raw umber pigment, blended with water, was applied on each sample in order to create painted specimens, having an area of (10 × 20) cm^2^. Moreover, in order to simulate a lack of cohesion of the paint layer, the application of the pigment was performed after 3 h from the spreading of the plaster layer so that the pigment grains could not be completely fixed to the mortar surface during the carbonatation process. Similar samples were prepared for efficacy and colorimetric tests (C and D, respectively), except for the fact that the paint layer of the specimens was realized with red ochre.

For what concerns the choice of the best application procedure of the NanoLAQ dispersions, different procedures were considered. Specifically, the ions’ contents and pH tests were carried out by applying the NanoLAQ10 dispersion, while for efficacy and colorimetric tests different application procedures were considered, increasing the amount of the consolidant product or alternating different concentrations, as reported in Table 1. For all the procedures, the product was applied by brush through a sheet of Japanese paper. Moreover, the application of water alone was considered in order to check the influence of the dispersing medium on the results.

After the laboratory analysis, the final tests were performed in situ, considering the application procedure that had given the best results during the laboratory step. The product efficacy was investigated on sampling areas of the paintings characterized by different color and decohesion degrees. The tests carried out in situ have been also repeated after 4 months, in order to evaluate the treatment efficacy over time.

Laboratory Tests: Investigation Procedures

#### 2.3.1. Ions’ Content Variation in Specimens

Some conductivity tests were made to verify the influence of the aqueous nanolime application on the ions’ content of the surface; also, regarding the chlorides content coming from the nanolime product.

The measurements were carried out by a Water Quality Meter AZ 836, by the following procedure:

(a) distilled water was spread on a (5 × 5) cm^2^ Japanese paper sheet, put on the mortar sample;
(b) the Japanese paper was not removed until drying;
(c) once dried, the Japanese paper was immersed in 50 mL of distilled water to measure the electrical resistance of the resulting solution, in order to verify the presence of possible soluble salts extracted by the Japanese paper.

The comparison was made between the conductivity values before and after nanolime treatment.

Some mortar specimens were treated by applying NanoLAQ10 according to the n. 01 procedure reported in Table 1. On the treated samples, the conductivity measurements have been carried out after 7 days from the application of the consolidant product in order to be sure of the complete carbonation process and avoid the contribution of calcium hydroxide to the conductivity value.

The measurements were carried out also on untreated specimens in order to verify if a conductivity variation was due to soluble salt present in the specimen itself and brought to the surface by water evaporation, independently from the application of the nanolime suspension.

#### 2.3.2. pH Test for the Evaluation of the Carbonatation Degree over Time on Specimens

In order to evaluate the carbonatation degree of the aqueous nanolime after its application over time, pH measurements of the painted surface of fresco-painted specimens, specifically prepared in laboratory, have been performed. Before and during the carbonatation test, the fresco specimens were conditioned at a temperature of 20 °C and relative humidity conditions between 96% and 98% to simulate the microclimatic conditions of the hypogeum and to see how they could affect the carbonatation process.

In these environmental conditions, each sample was divided in two portions: one portion was left untreated, while the other one was treated by NanoLAQ10, applied by brush through a sheet of Japanese paper. In particular, the comparative pH value of the untreated surface was named as **pH_thresold_**.

The first pH measurement on the treated areas was performed suddenly after the application of the product, while the subsequent measurements were performed at intervals of one hour between each other.

For all the measurements, a Flat Tip pH Electrode (*Hanna Instruments*^TM^, Italia srl, Ronchi di Villafranca Padovana (PD), Italy) was used.

#### 2.3.3. Efficacy Tests on Specimens

The efficacy of the different application procedures and concentrations of the aqueous nanolime, shown in Table 1, were tested in relation to the increase in the pigment cohesion on the substrate before and after the nanolime treatment. For this aim, the efficacy of a consolidating product is generally performed adapting the procedure described in ASTM D4214-07R15—*Standard Test Methods for Evaluating the Degree of Chalking of Exterior Paint Films*—generally known as scotch tape test (STT). STT consists of testing the cohesion of a surface by measuring the material removed by a pressure-sensitive tape, previously applied onto the surface [36]. Unfortunately, if STT can be adequately useful to measure the superficial cohesion of natural stones and historical mortars, it is extremely critical in the case of paint layers. In fact, when dealing with original historical paintings, STT presents two main issues: the first one is the highly destructive impact on the original surface, and the second one is the strong dependence of the test results on the operator ability and bias (particularly when there is not the possibility to carry out many samplings, statistically reliable) [37].

Then, considering the crucial problem relating to the evaluation of the test efficacy of a consolidant product on a historical paint layer, a new method is here proposed for the first time to be applied both on lab specimens and on site. This method, developed by the restorer of the ICR/Mural Painting Laboratory, allows to enhance the test reliability and repeatability and to limit the destructive action of the tape test on the original painting. In fact, the area investigated by the instrument can be very small, consisting of a circular area of 4 mm in diameter.

**Figure 1:**
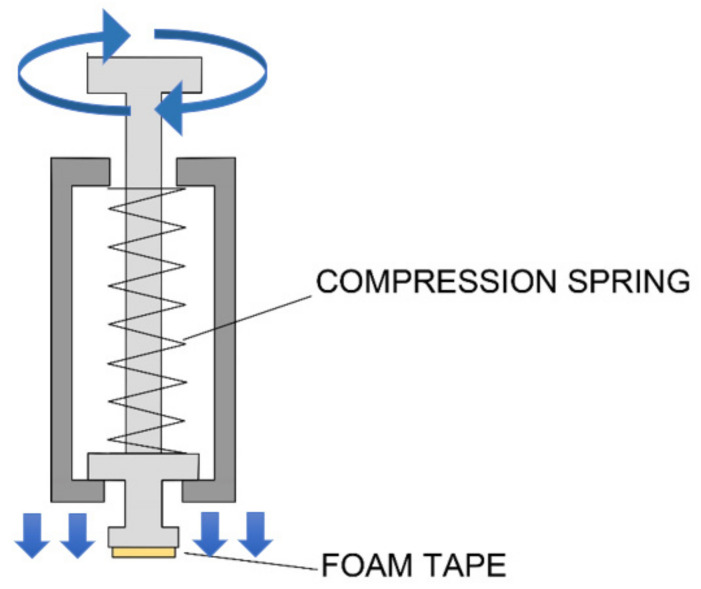
Spring instrument for measuring the decohesion degree of the paint layer.

This new method consists of performing the test by a self-made instrument, which is here named “spring instrument” (Figure 1), constituted by a cylindrical structure, containing a small piston which can vertically move along the axis of the cylinder. Around the piston, a compression spring is located which allows a fixed vertical pressure to be performed on the surface, while at the end of the piston a flat circular tip is fixed where an adhesive white foam tape can be applied and changed after each measurement. Besides the vertical pressure, the piston can rotate by an angle, established by the operator, up to covering a 360° rotation along the piston axis. This feature gives the possibility to apply a small and constant pressure, parallel to the paint layer, that lets the operator test the cohesion of the pigments, always using the same pressure and the same rotatory movement. This is also applicable in the case of wet surfaces where the scotch tape cannot adhere. The spring system assures repeatability and a comparison between different measurements, while the foam tape allows the testing tip to adhere perfectly to the roughness of the surface, making the measurement more accurate in the case of coarse surfaces.

During the measurement, if the pigment’s grains do not adhere to the substrate, they are rubbed away releasing a trace of color on the white foam tape. The amount of removed pigment can be quantified not only by a visual investigation but also by digital measuring by means of a photo-editing and raster graphic design software (*Adobe Photoshop CC*). In particular, the amount of pigment particles removed by the foam tape was analyzed and measured by the photo-editing software, through the following steps:

- to select and to isolate the areas of the circular foam tape colored by the pigment;
- to measure the loss of brightness due to the presence of different quantities of pigment particles on the white foam tape. The measure was carried out by converting the intensity of the coloration in a gray scale and measuring the “mean gray value” (referred to as “brightness”) of each area. The mean gray value represents a measurement of brightness, having values from 0 to 255 for 8-bit images.

In the tests here presented, three measurements were carried out on each specimen, considering different areas of the surface in order to obtain an average evaluation of the decohesion degree of the painted layer.

#### 2.3.4. Colorimetric Test on Specimens

The colorimetric test was performed to detect if any whitening of the surface occurred after the carbonatation of the NanoLAQ dispersion. The specimens used for the colorimetric test were the same used for the efficacy test. On each specimen, colorimetric measurements were carried out before and after 10 days since the nanolime treatment. The colorimetric values of the treated surface were compared with untreated samples.

Specifically, the measurements were carried out according to standard test method UNI 15886:2010—Conservation of cultural property—Test methods—Color measurement of surface [38]. A Minolta^®^ CM700d spectrophotometer (Konica Minolta Sensing Europe B.V., Cinisello Balsamo (MI), Italy) was used, with a daylight illuminant D65 and 10° observer, excluding the specular component. Considering the inhomogeneity of the surface, the diameter of the measuring area was 8 mm and the number of acquisitions of the tristimulus coordinates was set to five for each specimen. The final value was the average of the five acquisitions. Chromatic coordinates were elaborated by CIE L*a*b*76 system, giving the color differences in the surface before and after treatment, expressed by a numeric value, called **deltaE**.

In order to determine if water could cause any chromatic change to the painted surface, some specimens were treated only by applying pure water by brush through a sheet of Japanese paper.

### 2.4. In Situ Tests: Investigation Procedures

The same tests performed during the laboratory step were also carried out in situ on one of the mural paintings of the hypogeum in Matera, selecting the application procedure that gave the best results during the lab testing (procedure 04) on untreated and treated areas with the NanoLAQ dispersion.

**Figure 2:**
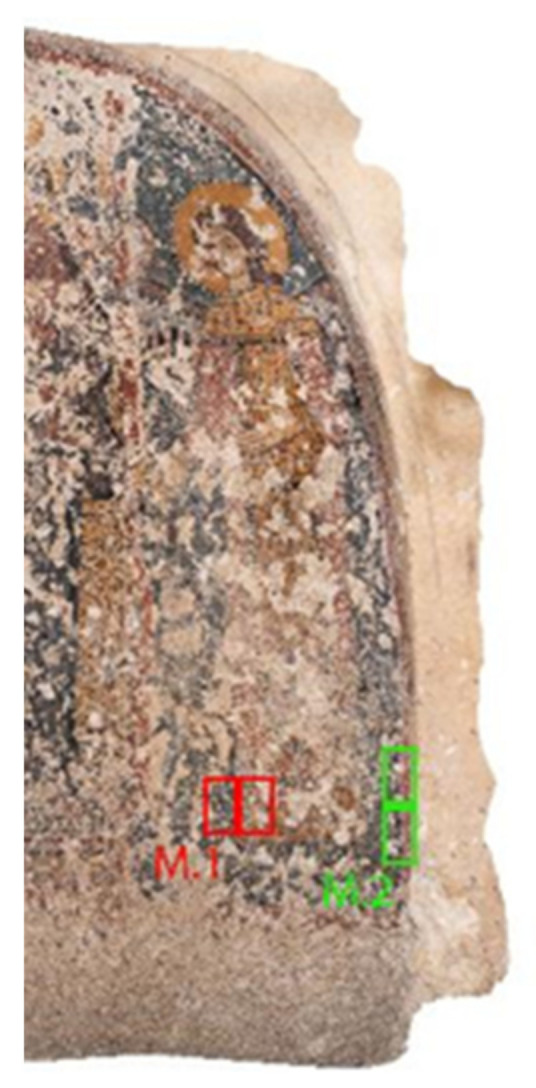
Conductivity measurements: two investigated areas of paintings in situ (M1 and M2 sample areas).

**Figure 3:**
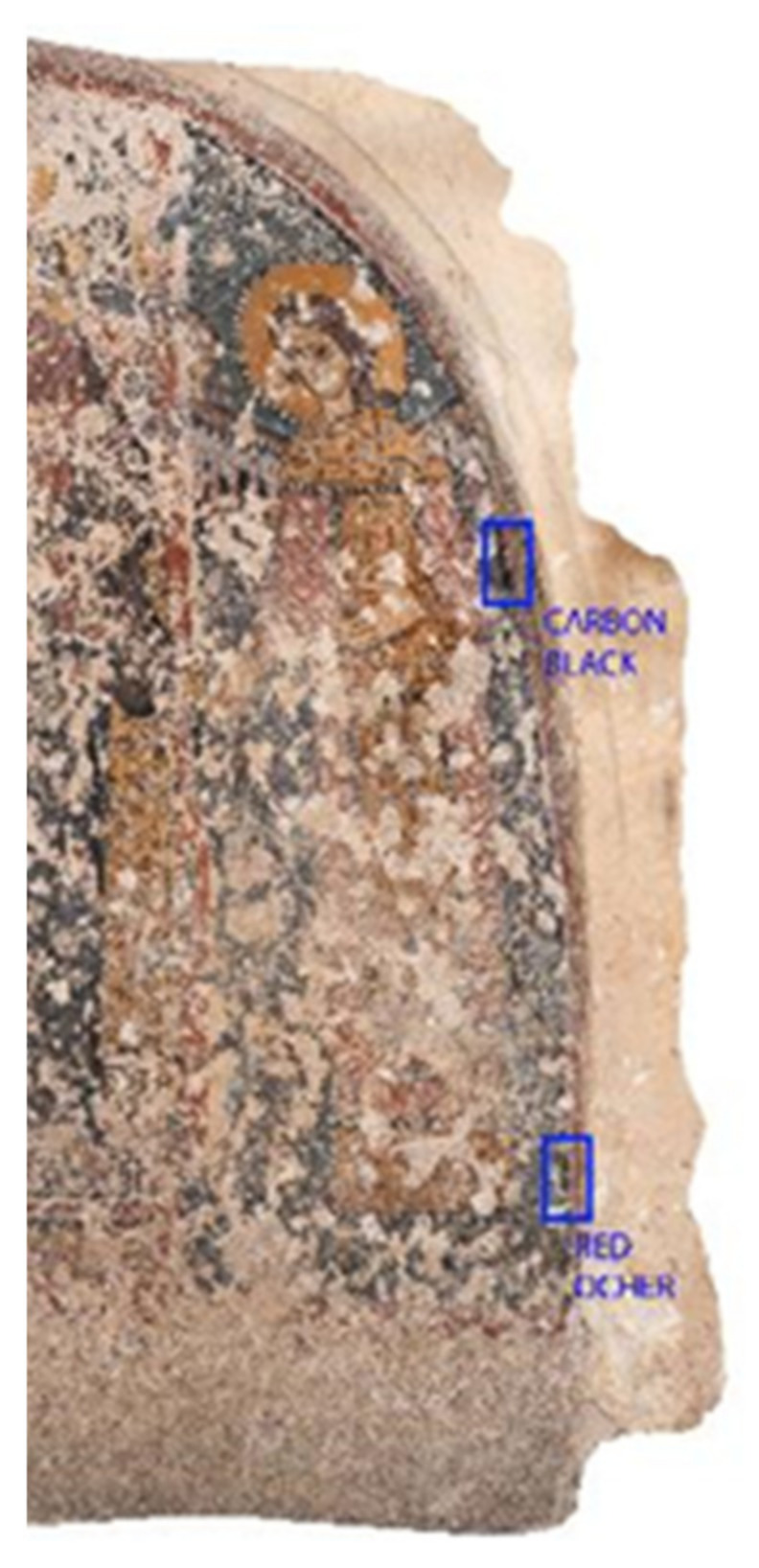
Sample areas of pH, efficacy, and colorimetric tests.

- The conductivity measurement was performed in the area shown in Figure 2.

- PH, efficacy, and colorimetric measurements were performed in relation to two different pigments of the fresco: carbon black and red ocher, as shown in Figure 3.

Finally, during in situ testing, NanoLAQ300 was applied to treat defects of adhesion between the paint layer and the plaster, evaluating the ability of the concentrated dispersion to be injected under the flakes of the paint film in order to create a filling layer and assure a good adhesion degree of the flakes.

## 3. Results and Discussion

### 3.1. The Aqueous Nanolime Suspension NanoLAQ

**Figure 4:**
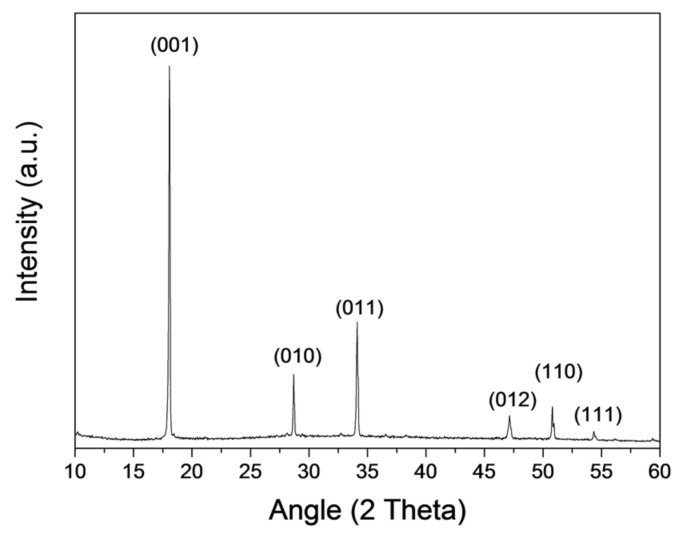
XRD diffraction pattern of the NanoLAQ suspension sample after drying under nitrogen atmosphere. The Bragg peak is indexed by the Miller index of Portlandite (ICDS #98-020-2220).

The synthesis allowed for the producing of, after the separation of the aqueous suspension from the resin, a dispersion of pure and crystalline Ca(OH)_2_ nanoparticles, as investigated by XRD, SEM, and TEM analyses. Specifically, from XRD results, only the presence of hexagonal portlandite is observed, with any secondary crystalline phases or amorphous contributions, as shown in Figure 4.

**Figure 5:**
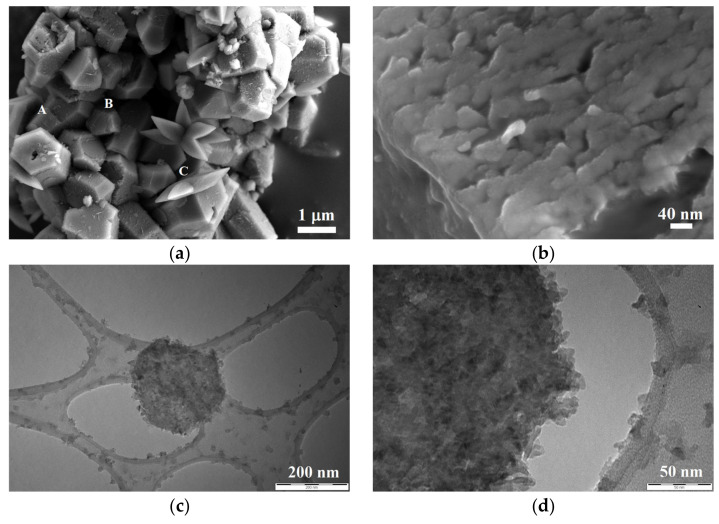
SEM and TEM observations of the NanoLAQ suspension sample after drying under nitrogen atmosphere: (**a**) low magnification SEM image showing the crystalline prismatic (A and B) and scalenohedral (C) habits present in the sample after drying on the SEM specimen stab; (**b**) high magnification SEM image of the surface of a prismatic crystal indicating that each crystal was composed of a dense nanoparticles aggregation; (**c**,**d**) TEM images representing a hexagonal aggregate composed of nanoparticles all ≤10 nm in size.

SEM investigation allowed the observation of the sample in relation to the morphology of the aggregates (lower magnification images), as well as of the single nanoparticles (higher magnification images). In this regard, in Figure 5a, several prismatic crystals of dimension around 1 µm were observed, marked as A and B, matching the typical crystalline habits of portlandite. Moreover, probably due to an initial carbonatation occurring during the sample drying on the SEM specimens stab, the presence of some crystals of calcite was observed too, according to a scalenohedral crystal habit (marked by the C letter), as also observed in a previous work [28]. If observed at higher magnification (Figure 5b), each prismatic crystal appeared composed by a dense distribution of nanoparticles less than 20 nm in size. This observation was confirmed by TEM images (Figure 5c,d), which underlined the presence of nanoparticles of 10 nm in size or less, having the tendency to ordinately assembly in a hexagonal aggregate, the typical Ca(OH)_2_ crystalline structure.

Concerning the chlorides measurements, after the synthesis, a residual content of only 100 mg/L was obtained. However, considering the specific need of operating on mural paintings, a washing was carried out, which left in the NanoLAQ suspension a residual chloride content of 15 mg/L.

### 3.2. Laboratory Test Results

#### 3.2.1. Ions’ Content Variation in Specimens: Results

The results of the conductivity measurements showed very low values, with no significant variations in the conductivity before and after the treatment, as reported in Table 2, confirming that the quantity of soluble salts left in the porous system is negligible. Moreover, the observed conductivity values assured that the residual chloride content of the NanoLAQ product, left on the surface by following the application procedure 01, is negligible. In fact, all the conductivity values measured during the test are similar to that of demineralized water usually employed for conservation treatments on mural paintings.

#### 3.2.2. pH Test for the Evaluation of the Carbonation Degree over Time on Specimens: Results

**Figure 6:**
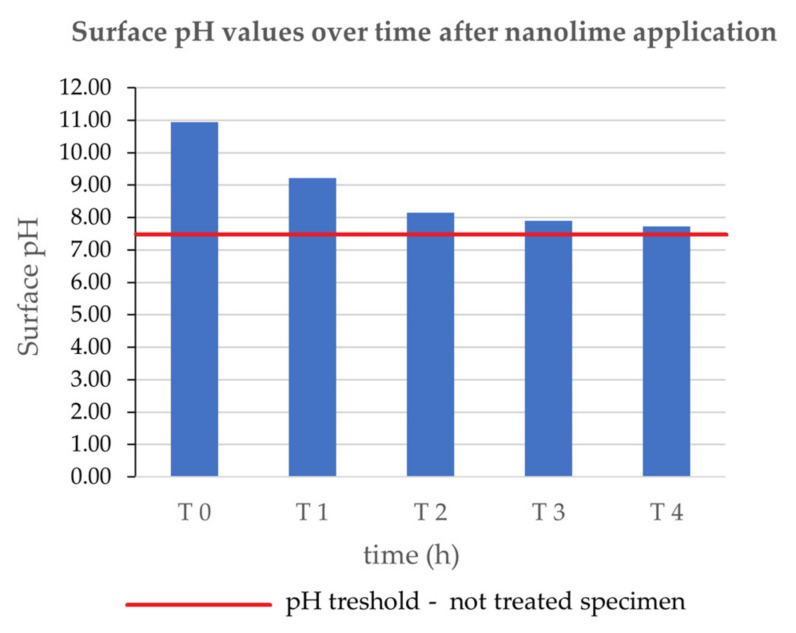
pH values over time on specimens. The red line represents the pH value of the untreated specimens (**pH_threshold_**).

The measurements of the superficial pH showed that the surface reached a complete carbonation degree in a short time. Actually, the superficial pH values of treated specimens varied from a value of 11.03 at T0 to neutral values after only 4 h from the treatment application, reaching the same pH values of the untreated surface (see Figure 6). This result can represent a very important performance of the aqueous nanolime dispersion NanoLAQ, assuring the first necessary condition for the efficacy of the consolidating action of the treatment.

#### 3.2.3. Efficacy Tests on Specimens: Results

The efficacy test was performed after 24 h from the treatment application. The results are shown in Table 3, where the visual observations and the digitally elaborated results are reported in correspondence to each application procedure. We can observe that already for the lowest amount of NanoLAQ applied to the substrate (two applications of NanoLAQ10), an increase in the cohesion of the paint layer was obtained. In fact, the amount of pigment particles rubbed away by the spring instrument from the surfaces treated with two applications of NanoLAQ10 are considerably less than that removed from untreated paint layers. Moreover, there are no remarkable differences between the different procedures of NanoLAQ tested; although, a slightly better cohesion is obtained when the treatment procedure involves the use of NanoLAQ20 in addition to NanoLAQ10 (04 procedure), as shown from the digitally elaborated results. This result can be attributed to a different morphology in the calcite crystals resulting from the carbonation process when a higher concentration of Ca(OH)_2_ is used. In fact, in this case, the carbonatation of the aqueous nanolime dispersion can lead to the formation of a more remarkable growth of scalenohedral calcite crystals with respect to the product having a concentration of 10 g/L, as discussed in a previous paper [28], probably due to the coexisting effect of both a high concentration and high-water content during the carbonatation process.

#### 3.2.4. Colorimetric Test on Specimens: Results

The colorimetric analysis of the specimens prepared and treated in the laboratory is reported in Table 4. We observed that the procedures involving the use of only NanoLAQ10 have **deltaE** values relatively low, similar to the values observed when water alone was applied. Instead, all the procedures involving the application of NanoLAQ20 caused a slight whitening of the surface, although the **deltaE** values can be always considered adequate [39]. In addition, regarding the analyses of the single parameters, the L* and a* parameters remained almost unvaried in each specimen, while b* underwent a more consistent variation, indicating a general shift of the color from the yellow to the blue component. This behavior was quite unexpected, and it is probably due to the saturating and compacting action of water and brush pression on the incoherent grains of pigment, as confirmed by the **deltaE** values measured for the specimen treated with water alone.

### 3.3. In Situ Tests: Results

As previously stated, only the application procedure 04 (that is, two applications of NanoLAQ10 + one application of NanoLAQ20), which exhibited the best results in laboratory tests, was considered for the in situ tests on paintings. Here, except for the pH measurement, all the tests have been performed both right after the NanoLAQ applications (referred to as T0) and after 4 months (referred to as T4).

From the analysis in situ, it can be observed that some little variations in the conductivity value were measured, probably due to an intrinsic inhomogeneity of the substrate and of the salts naturally contained in the plaster. Actually, there are no significant variations in the conductivity values of treated and not treated areas (see Table 5), both when the measurements were carried out soon after the application, without transport of ions on the surface, confirming the results obtained in the laboratory and after 4 months. This also means that the content of chloride ions remains negligible also when the product is applied following application procedure 04, despite the higher content of product put on the surface if compared with procedure 01.

**Figure 7:**
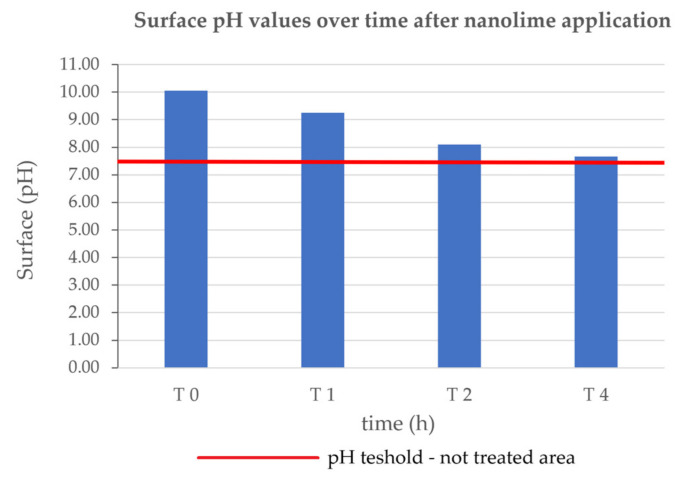
pH values over time (in hours) on paintings in situ.

Regarding the pH measurements, tests performed in situ demonstrated that a good carbonatation degree was reached after 4 h from the application of the product, as also observed in the laboratory specimens (see Figure 7*)*.

After 24 h from the treatment, the efficacy test was performed, using the spring instrument previously described. The paint layer showed a greater level of cohesion if compared with the untreated areas, both on red ochre and carbon black colors (see Table 6), underlying the presence of the new network of calcite crystals deriving from the carbonatation of NanoLAQ.

The same test repeated after 4 months from the application gave similar results, showing a level of cohesion of the paint remained almost unvaried if compared to that obtained 4 h after the treatment (see Table 6), a fundamental result which assures the consolidating stability of the new calcite crystals over time

The colorimetric test was performed on treated and untreated areas of the paintings in situ on different colors: red and black. The measurement was performed 24 h after the treatment when the carbonation process had already occurred, as highlighted by the other tests, and then it was repeated after 4 months from the treatment in order to verify if any chromatic change had occurred meanwhile. The results, reported in Table 7, showed only slight differences in the chromatic values suddenly after the treatment between treated and untreated areas, with **deltaE** values always below the value of three.

The same test repeated after 4 months demonstrated that no other chromatic change in the surface had occurred over time: **deltaE** values obtained could be comparable to that due to the inhomogeneity of the paint layer itself.

**Figure 8:**
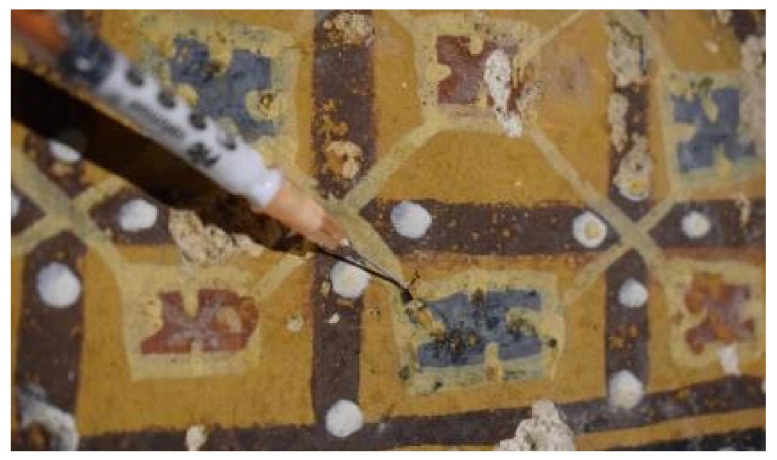
Treatment of defects of adhesion of the paint layer with NanoLAQ300 by means of a syringe.

Finally, the in situ use of NanoLAQ300 as filler for detachments of the paint layer gave good results. The concentrated dispersion showed good easy-handling properties when injected by means of a syringe, better than that of pure lime paste, and assured a good adhesion of the flakes in a short time (Figure 8). The use of nanolime to treat defects of adhesion of the paint layer allows restorers to avoid the use of traditional adhesive products made of acrylic compounds, which could cause a detrimental localized reduction in the permeability of the treated areas. The evaluation of the adhesion degree of the flakes was performed empirically, thus needing further laboratory evaluation to be carried out in following studies.

## 4. Conclusions

The innovation of this study is mainly related to an applicative field, as this study had the purpose of suggesting a solution to a difficult and unsolved issue concerning the consolidation of mural paintings in the hypogeum environment. These environments are characterized by high levels of relative humidity and low air circulation. This condition made it inadvisable to use organic consolidants, such as acrylic emulsions, particularly sensitive to biodegradation, as well as consolidant products dispersed in or producing alcohols, which could favor the growth of biodeteriogen colonization and represent a considerable health issue for operators in this kind of environment.

For these reasons ICR restorers decided to test a new nanolime dispersion, NanoLAQ, constituted by a pure dispersion of calcium hydroxide nanoparticles in water and obtained by an innovative, sustainable, and large-scale production process, used for the first time on a mural painting in hypogeum in this application study. Thanks to the presence of water as a dispersing medium and the absence of any organic additive, it resulted to be perfectly compatible and potentially suitable to solve a problem which is still one of the most complicated issues in the restoration of hypogeic cultural heritage. The NanoLAQ water dispersion underwent several tests, carried out first in a laboratory and then in situ, in order to evaluate its suitability and efficacy to be used as consolidant of the paint layer of some rupestrian frescoes *frescoes* in SS. Peter and Paul’s hypogeum in the UNESCO site of Matera (Italy). The measurements of the ions’ content of the surface, performed in order to determine whether the residual chloride content of the product could affect the soluble salt content of the surface, showed that no significant variation occurred after the application of the product, independently from the quantity of NanoLAQ applied on the surface. PH measurements, performed in order to control completeness of the carbonation degree in such a humid environment (90% UR), showed that the neutralization of the superficial pH and then the carbonatation occurred within 4 h from the application of the nanolime product, thus confirming the particular suitability of this product for hypogeum environments. The time interval for the completion of the carbonation process was the same, both for the laboratory specimen and in situ application of the product. The efficacy tests, related to the ability of the product to re-establish the cohesion of the pigment, were performed by using a new instrument, here introduced and experimented for the first time and named “spring instrument”. The spring instrument allows to enhance the reliability and the repeatability of the measurement if compared to the traditional scotch tape test, providing a minimized destructive action towards painted layers. The tests, first performed on laboratory specimens and then in situ, showed a good ability of NanoLAQ products, independently from the number of applications and concentration of the suspension, to reduce the decohesion of the paint layers. The tests, moreover, were repeated after 4 months since the treatment, revealing that the same level of cohesion was maintained by the paint layer over time.

Finally, colorimetric measurements, performed to verify if any chromatic change in the surface occurred after the application of the product, showed that a negligible whitening of the surface occurred also when a high concentration of NanoLAQ was considered. The in situ colorimetric test showed chromatic alterations lower than those detected for lab specimens, which were stable over time.

In addition to the good results of the in situ application tests, it was observed that no alteration of the surface due to biodeteriogen degradation occurred after four months since the application of the product, as expected, confirming the potential suitability of this product for its use in hypogeic environments.

Considering the positive results of the preliminary tests, during the restoration of the medieval mural paintings of the SS. Peter and Paul Hypogeum, a wider sample area of the painting was selected to apply the product more extensively. The consolidation treatment with NanoLAQ let restorers carry out the cleaning procedure without any risk for the paint layer because the cohesion of the paint was previously re-established.

During the restoration, the same product was used also to treat defects of adhesion between the paint layer and the plaster by injecting NanoLAQ300 (300 g/L aqueous nanolime dispersion) under the flakes of the paint layer, avoiding the use of traditional acrylic resins.

A monitoring program has been designed and is going to be carried out to monitor the product behavior over a longer period of time. At the same time, further analytical studies are being carried out by researchers of the two institutions to verify the suitability and the effectiveness of this product over time, performing different application tests in other hypogeic restoration sites. 

## Figures and Tables

**Table 1:** Application procedures and concentrations considered in the laboratory tests.

Label	Procedure	Tests Performed in Lab	Tests Performed In Situ
0	Not treated	C, D	-
00	3 applications of water	C, D	-
01	2 applications NanoLAQ10	A, B, C, D	-
02	3 applications NanoLAQ10	C, D	-
03	4 applications: NanoLAQ10	C, D	-
04	2 applications NanoLAQ10 + 1 application NanoLAQ20	C, D	A, B, C, D
05	1 application NanoLAQ10 + 2 applications NanoLAQ20	C, D	-

Legend: A: ions’ content test; B: pH test; C: efficacy test; D: colorimetric test.

**Table 2:** Values of the conductivity before and after the treatment performed by using nanolimes.

Conductivity (µS/cm)		
	Before Treatment	After Treatment
Control specimen	2	3
Treated specimen, procedure 01	2	8

**Table 3:** Results of the efficacy tests carried out on the specimens prepared in laboratory.

	Visual Circular Foam Tape	Brightness
0	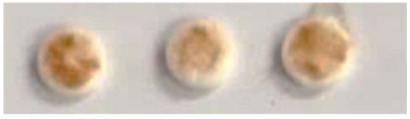	114
00	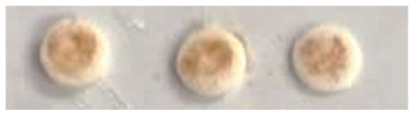	125
01	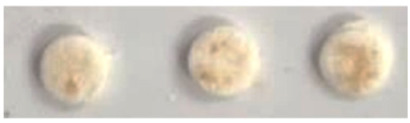	136
02	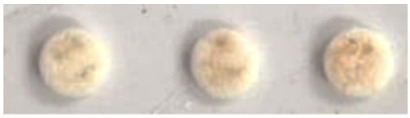	136
03	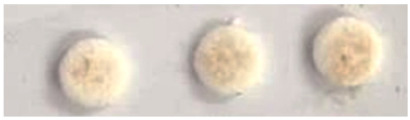	139
04	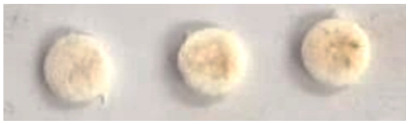	140
05	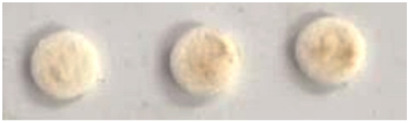	139

**Table 4:** The results of colorimetric test for the evaluation of chromatic changes after the application of different nanolime treatments.

Treatment	Specimen	L	a*	b*	Delta E
0	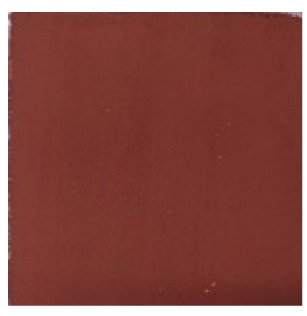	38.8	24.8	24.5	-
00	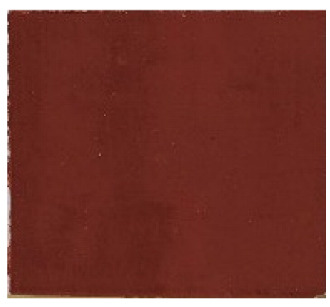	36.8	24.7	23.7	2.1
01	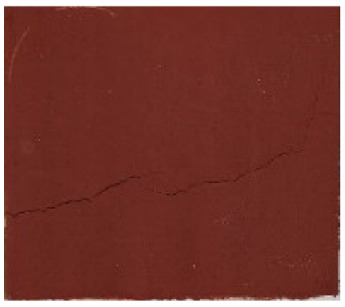	37.5	25.0	23.3	1.8
02	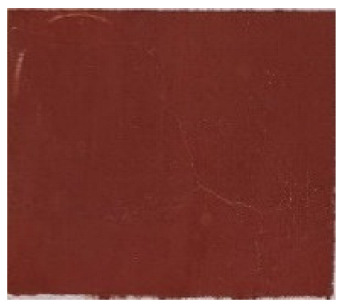	38.3	24.4	22.4	2.2
03	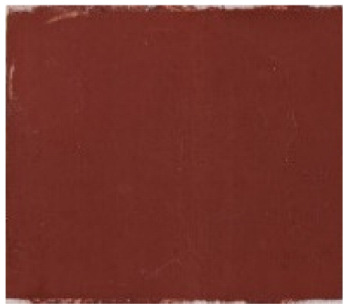	39.1	24.1	22.0	2.6
04	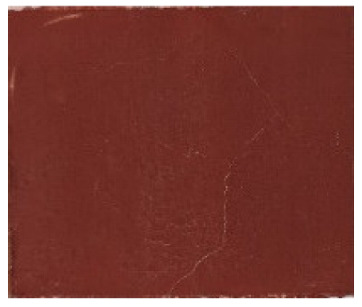	38.7	23.9	21.6	3.0
05	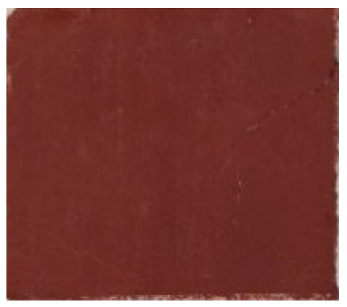	38.3	23.6	21.3	3.5

**Table 5:** Values of the conductivity before and after the consolidation treatment with aqueous nanolime in situ.

Point of Measurement	Untreated	Treated T0	Treated T4 Months
M. 1	21 µS/cm	12 µS/cm	18 µS/cm
M. 2	5 µS/cm	9 µS/cm	5 µS/cm

**Table 6:** Efficacy test on paintings in situ.

Color	Treatment	Time from the Application	Efficacy Test	Brightness
Carbon black	Untreated		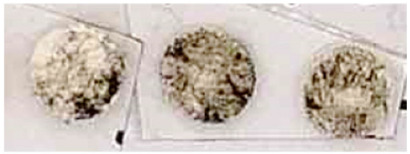	81
	2 applications NanoLAQ10 + 1 application NanoLAQ20	T0	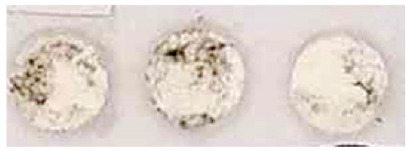	119
		T4 months	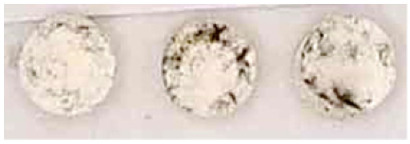	117
Red ochre	Untreated		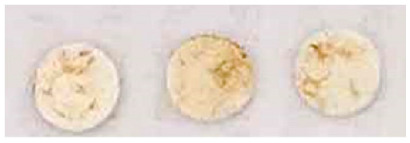	121
	2 applications NanoLAQ10 + 1 application NanoLAQ20	T0	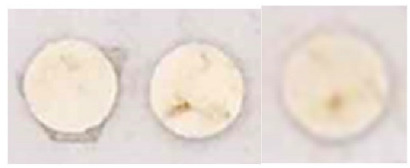	131
		T4 months	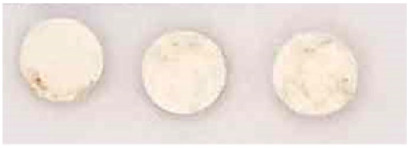	136

**Table 7:** DeltaE values of treated and untreated areas of paintings in situ.

Color of the Paint Layer	Treatment	Delta E T0	Delta E T4 Months
Red ochre	2 applications NanoLAQ10 + 1 application NanoLAQ20	0.28	0.71
Carbon black	2 applications NanoLAQ10 + 1 application NanoLAQ20	2.36	1.45

## Data Availability

Not applicable.
